# The serum acylcarnitines profile in epileptic children treated with valproic acid and the protective roles of peroxisome proliferator-activated receptor *a* activation in valproic acid-induced liver injury

**DOI:** 10.3389/fphar.2022.1048728

**Published:** 2022-11-08

**Authors:** Yiyi Ma, Minglu Wang, Shuaishuai Guo, Tong Li, Xiaodong Liu, Limei Zhao

**Affiliations:** Department of Pharmacy, Shengjing Hospital of China Medical University, Shenyang, China

**Keywords:** valproic acid, acylcarnitines, fatty acid β-oxidation, peroxisome proliferator-activated receptor α, hepatotoxicity

## Abstract

Valproic acid (VPA) is widely used as a major drug in the treatment of epilepsy. Despite the undisputed pharmacological importance and effectiveness of VPA, its potential hepatotoxicity is still a major concern. Being a simple fatty acid, the hepatotoxicity induced by VPA has long been considered to be due primarily to its interference with fatty acid β-oxidation (β-FAO). The aim of this study was to investigate the biomarkers for VPA-induced abnormal liver function in epileptic children and to determine potential mechanisms of its liver injury. Targeted metabolomics analysis of acylcarnitines (ACs) was performed in children’s serum. Metabolomic analysis revealed that VPA -induced abnormal liver function resulted in the accumulation of serum long-chain acylcarnitines (LCACs), and the reduced expression of β-FAO relevant genes (Carnitine palmitoyltrans-ferase (CPT)1, CPT2 and Long-chain acyl-CoA dehydrogenase (LCAD)), indicating the disruption of β-FAO. As direct peroxisome proliferator-activated receptor *a* (PPARα)- regulated genes, CPT1A, CPT2 and LCAD were up-regulated after treatment with PPARα agonist, fenofibrate (Feno), indicating the improvement of β-FAO. Feno significantly ameliorated the accumulation of various lipids in the plasma of VPA-induced hepatotoxic mice by activating PPARα, significantly reduced the plasma ACs concentration, and attenuated VPA-induced hepatic steatosis. Enhanced oxidative stress and induced by VPA exposure were significantly recovered using Feno treatment. In conclusion, this study indicates VPA-induced β-FAO disruption might lead to liver injury, and a significant Feno protective effect against VPA -induced hepatotoxicity through reversing fatty acid metabolism.

## Graphical Abstract

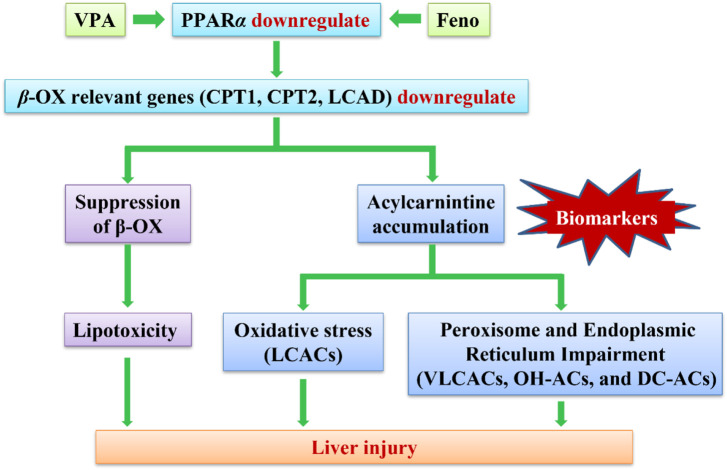

## 1 Introduction

VPA is a broad spectrum antiepileptic drug that is mainly used in the treatment of epilepsy and bipolar disorder. In recent years, as a histone deacetylases (HDACs) inhibitor, it has been approved for being an adjunct to cancer therapy (Heers et al., 2018). However, unintended adverse drug reactions limit its clinical use, especially hepatotoxicity, which is the most common and life-threatening one (Meijboom and Grootens, 2017). Studies have shown that hepatotoxicity induced by VPA may be related to secondary carnitine deficiency (Lheureux and Hantson, 2005). Carnitine deficiency has been found in patients treated with long-term VPA(Qiliang et al., 2018). Intravenous carnitine supplementation has been reported to treat severe and symptomatic hepatotoxicity caused by VPA (Felker et al., 2014).

The mechanism of hepatotoxicity caused by VPA has been reported in many studies (Bűdi et al., 2015; Komulainen et al., 2015; Katayama et al., 2016; Ahangar et al., 2017), but the exact mechanism remains to be under-elucidated. Hepatotoxicity due to VPA is associated with carnitine deficiency and abnormal β-FAO of fatty acids. VPA causes carnitine deficiency *via* different mechanisms (Ishikura et al., 1996; Gidal et al., 1997; Matsumoto et al., 1997; Raskind and El-Chaar, 2000). The depletion of carnitine and free CoA impair the transport of fatty acids into the mitochondria, resulting in elevated blood lipid levels, as well as lipid deposition and steatosis in hepatocytes.

When an impairment in β-FAO pathway of fatty acids occurs, it causes the accumulation of the corresponding acyl-CoAs, resulting in an alteration of the corresponding ACs in plasma. In contrast to acyl-CoAs, which remain sequestered in mitochondria, ACs can be transported out of mitochondria and out of the cell to the blood-stream and thereby directly reflect the intracellular pool of corresponding acyl-CoAs(ter Veld et al., 2009). Currently, plasma ACs profiling has commonly been used for the diagnosis of mitochondrial fatty acid oxidation deficiencies (mFAODs) in neonates and is an important biomarker of mitochondrial dysfunction (Rinaldo et al., 2005) To study the effects of VPA on carnitine status and the metabolic pathways associated with β-FAO, analysis of ACs profiles is feasible.

Peroxisome proliferator-activated receptor alpha (PPARα) is a transcription factor that regulates transcription by binding to the PPARα response elements of several genes, including genes related to mitochondrial and peroxisomal β-FAO (Kersten and Stienstra, 2017). Several studies have shown that PPARα activation mediated by the PPARα agonist Feno promotes the expression of genes involved in hepatic β-FAO (Zhao et al., 2017)and limits hepatic steatosis associated with high-fat diet, T2DM and obesity-related insulin resistance (Kostapanos et al., 2013), nonalcoholic hepatitis (Kersten and Stienstra, 2017) and cholestatic liver disease (Ghonem et al., 2015).

In this study, we propose to investigate the possible mechanism of VPA-induced disorder of ACs profiles, relative gene expression, including ACs transport and β-FAO enzyme was tested at the cellular and animal levels to explore the candidate biomarkers for VPA-induced abnormal liver function. In addition, based on the activation of mitochondrial β-FAO metabolic pathways to find potentially protective agents of VPA induced abnormal liver function.

## 2 Methods

### 2.1 Clinical information and sample collection

The study involving human participants were reviewed and approved by the medical ethics committee of Shengjing Hospital of China Medical University (No. 2016PS43K). A total of 50 healthy children and 55 children with epilepsy (aged≤ 16 years) were enrolled in this study. All children with epilepsy were diagnosed with symptomatic epilepsy and treated with VPA for at least 2 months. For each child, age, gender, height, body weight, and liver function indicators, i.e., total protein (TP), albumin (ALB), alanine transaminase (ALT), aspartate transaminase (AST), glutamyltransferase (GGT), alkaline phosphatase (ALP), and total bilirubin (TBIL), were recorded. For epileptic children, VPA daily dose, duration of VPA treatment as well as VPA concentration were recorded simultaneously. According to the level of liver function index (ALT>2×Upper limit of normal and/or AST>2×Upper limit of normal) the epileptic children were divided into normal liver function group (NLF group) and abnormal liver function group (ALF group). All the data are shown in Supplementary Table S1.

### 2.2 Animals and treatment

The study protocol was reviewed and approved by the Animal Ethics Committee of China Medical University. (No. CMU2021302). Male C57BL/6J mice (aged 7 weeks) were purchased from Huafu Kang Biotechnology Co., Ltd. (Beijing, China). All mice were housed under SPF conditions with temperature control at 20°C–25°C and a 12 h light/dark cycle. All mice were allowed to eat and drink freely and had a 1-week acclimation.

Experiment 1 To investigate the expression of genes and proteins related to ACs in VPA-induced liver injury, all mice were randomly divided into 3 groups of 6 mice each. Mice in the experimental group were given 250 or 500 mg/kg VPA solution by gavage daily, and mice in the control group were given an equal volume of saline (5 ml/kg) for 28 days.

Experiment 2 To investigate the protective effect of PPARα on VPA-induced liver injury, all mice were randomly divided into 4 groups of 6 mice each. (1) control group; (2)Feno group; (3) VPA group; (4) VPA + Feno group. Feno and VPA + Feno groups were treated with Feno (50 mg/kg dissolved in 0.5% sodium carboxymethylcellulose (CMC-Na)) for 28 days; VPA and VPA + Feno groups were treated with VPA solution dissolved in saline for 28 days; control group were given an equal volume of saline for 28 days.

After the last administration all mice were fed with water only, and 24 h s later, mice were anesthetized by intraperitoneal injection of 1% sodium pentobarbital (50 mg/kg). Blood samples were collected into anticoagulated tubes, centrifuged at 1000 rpm for 10 min at 4°C, and the plasma samples were separated and stored in a −80°C refrigerator. The whole liver was removed, and the same part of the liver tissues were taken and fixed in 4% paraformaldehyde solution, and the rest of the liver tissues were stored in a −80°C refrigerator.

### 2.3 Cell culture and treatment

The human normal hepatic cell line LO2 (Shanghai Institute of Biological Sciences, Chinese Academy of Science, Shanghai, China) was maintained in DMEM(BI,United States) containing 10% FBS (Gibco, United States) and 1% penicillin/streptomycin (Gibco, United States) in a 5% CO2 humidified 37°C incubator. Fresh medium was added to cells every 2–3 days. Cells (2 × 10^6^) were treated with different concentrations of VPA and/or Feno for 24 h in 60-mm cell culture dishes. Then, supernatants were removed and dishes were washed with PBS three times. The cells were subjected to liver function index determination, oil red O staining, and mRNA or protein extraction.

### 2.4 UPLC-MS analysis of serum ACs and lipid

Serum sample pretreatment: Add 50 μl of waste serum samples after VPA blood concentration monitoring into the corresponding numbered centrifuge tubes, add 1 ml of lipid extract (including internal standard mixture); vortex for 2 min, sonicate for 5 min, add 200 μl of water; vortex for 1 min, centrifuge for 10 min at 12,000 r/min at 4°C; after centrifugation, aspirate 200 μl of supernatant into the numbered centrifuge tubes and concentrate; re-solubilize with 200 μl of lipid reagent and use for UPLC-QTOF/MS analysis.

Chromatographic conditions: ExionLC™ Ultra Performance Liquid Chromatography (UPLC) system (SCIEX Corp.), including liquid phase pump, integrated degassing device, autosampler, column temperature chamber, etc; Thermo Accucore™ C30 column (2.6 μm, 2.1 mm × 100 mm i. d.), column temperature 45°C.; Mobile phase: A-phase, acetonitrile/water (60/40, V/V) (containing 0.1% formic acid, 10 mmol/L ammonium formate); B-phase, acetonitrile/isopropanol (10/90, V/V) (containing 0.1% formic acid, 10 mmol/L ammonium formate); flow rate 0.35 ml/min; injection volume 2 μl.

The mass spectrometry conditions: High-resolution tandem mass spectrometry system SCIEX Triple TOF 6500+ with full scan in ESI ionization mode with positive and negative ion detection modes, respectively, in the TOF/MS scan range (m/z: 100–1000).

### 2.5 Biochemical and histopathological analysis

ALT, AST, ALP in mouse plasma were measured using the Catalyst One Chemistry Analyzer (IDEXX Laboratories, Westbrook, ME, United States). ALT, AST, ALP, in LO2 cell were monitored using assay kits (Nanjing Jiancheng Bioengineering Institute, Nanjing, China). GSH and MDA was measured by assay kits (Solarbio Science and Technology Co., Beijing, China). For histopathology, fixed liver tissues were dehydrated, embedded in paraffin, and sectioned at 4 μm for hematoxylin-eosin (H&E) staining. Oil Red O staining kit (Solarbio Science and Technology Co., Beijing, China) for lipid staining in LO2 cell.

### 2.6 Real-time PCR analysis

Total RNA was prepared from liver tissues and LO2 cells using the simple RNA Extract kit (Tiangen Biotech, China) and reverse transcription of RNA to cDNA using Fast King cDNA First Strand Synthesis Kit (Tiangen Biotech, China). Quantitative PCR (qPCR) was performed using Real-time quantitative PCR kit (TaKaRa, Japan) and an LightCycler^®^ 96 system (Roche, Switzerland). Melting curve analysis was performed to confirm the production of a single product in each reaction. Changes in gene expression were determined by normalizing mRNA levels to those of GAPDH as an internal control and fold change was calculated using the 2^−ΔΔCT^ method. Sequences of real-time PCR Primers are shown in Supplementary Table S2.

### 2.7 western blotting

Total proteins were extracted from liver tissues using RIPA buffer (Beyotime Biotech, China) supplemented with 1 mM PMSF (Beyotime Biotech, China). The membranes were blocked with 5% nonfat dry milk in PBS supplemented with 0.1% (v/v) Tween 20 and incubated overnight at 4°C with anti-CPT2, PPARα (Santa Cruz Biotechnology,United States), CPT1A, LCAD, Very long-chain acyl-CoA dehydrogenase (VLCAD), (Proteintech, United States), GAPDH (from Cell Signaling Technology, United States). Blots were developed with SuperSignal West Femto Maximum Sensitivity Substrate (Thermo Scientific). Immunodetection analysis was accomplished using an enhanced chemiluminescence solution (ECL, Millipore, United States). Band densities were quantified using ImageJ Software. The relative expression levels of purpose proteins were normalized to GAPDH levels.

### 2.8 Data analysis

The data were presented as the means ± SD or means ± SEM. Appropriate statistical analyses were performed using SPSS version 24.0 (SPSS, Chicago, IL, United States). Statistical analysis was performed using the one-way ANOVA or the two-tailed Student t-test. *p*-values of less than 0.05 were considered significant. Correlation factor (r) was estimated with Pearson’s correlation analysis.

## 3 Result

### 3.1 Analyses of serum ACs levels in VPA-induced abnormal liver function children samples

#### 3.1.1 Clinical characteristics of children

A total of 50 healthy children (Control group) and 55 children with epilepsy administered VPA were enrolled in this study (Supplementary Table S1). There were no statistically significant differences among the three groups in terms of age, sex, daily VPA dose, duration of treatment or VPA serum concentration. In the ALF group, the levels of ALT, AST, ALP and GGT were significantly higher than those in the other two groups.

#### 3.1.2 Analysis of ACs species in the three groups of children

##### 3.1.2.1 ACs species accumulate markedly in VPA-induced abnormal liver function children samples

**FIGURE 1:**
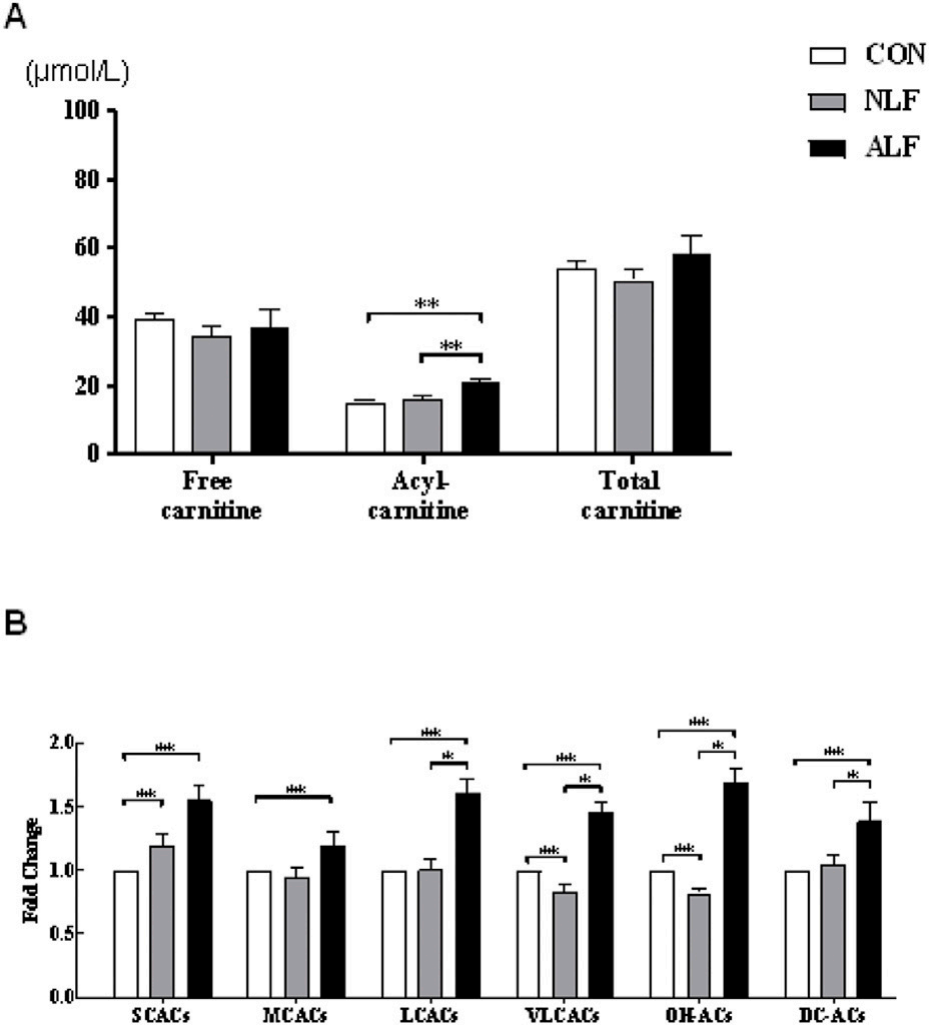
Altered ACs profile in three groups of children. **(A)** Changes in free carnitine, ACs, and total carnitine levels. (means ± SE) **(B)** Changes in the levels of different types of ACs. (means ± SD).*p < 0.05, **p < 0.01.

**FIGURE 2:**
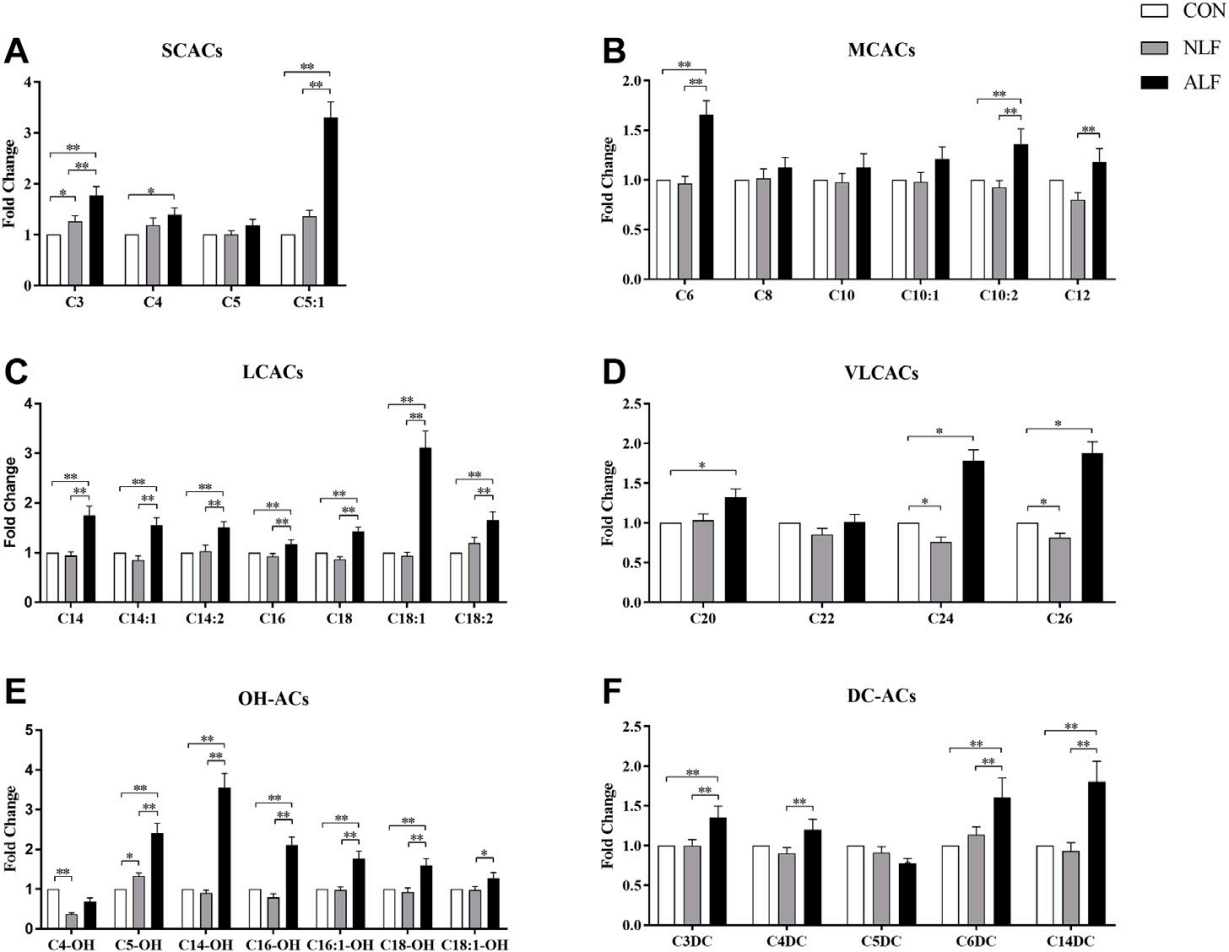
Differences in the 35 ACs levels among three groups of children. (means ± SD) Cx refers to the number of carbons in the acyl chain of carnitine derivatives. *p < 0.05, **p < 0.01.

A targeted metabolomic analysis of free carnitine and the 35 ACs species was performed using liquid chromatography-mass spectrometry (LC-MS). The various types of ACs are classified as follows: acetylcarnitines (C2), short-chain ACs (SCACs: C3+C4+C5), medium-chain ACs (MCACs: C6+C8+C10 + C12), long-chain ACs (LCACs: C14 + C16 + C18), very long-chain ACs (VLCACs: C20 + C22 + C24 + C26), hydroxylated ACs (OH-ACs) and dicarboxylic acid ACs (DC-ACs). Cx refers to the number of carbons in the acyl chain of carnitine derivatives. Summary statistics of the concentrations of free carnitine and different species of ACs in the three groups are provided in Figure 1 and Figure 2. As shown in Figure 1A, concentrations of ACs were significantly increased in NLF group andALF group. (*p* < 0.01), while free carnitine and total carnitine did not show any difference among the three groups (*p* > 0.05). Pairwise comparisons showed that all species of ACs were increased in ALF groups compared with the control (*p* < 0.01). However, LCACs, VLCACs, OH-ACs and DC-ACs were specifically accumulated in ALF group compared with NLF group (Figure 1B).

A separate analysis of specific ACs in the three groups are provided in Figure 2. Among the SCACs and MCACs, concentrations of C3-, C5:1-, C6-, C10:2- and C12-ACs differed among the three groups (*p* < 0.05). Additionally, ALF group had mean peak levels of C5:1-AC that were >5-fold higher than the control group. As shown in Figure 2C, all species of LCACs were significantly increased in ALF groups compared with both control and NLF group. Expect these standard LCACs such as C16-,C18:1- and C18:2-ACs, less usual ACs such as C20-, C24-, and C26-ACs (Figure 2D) as well as some OH-ACs (C5-OH-,C14-OH-, C16-OH- and C18-OH-ACs; Figure 2E) and DC-ACs (C3-DC-,C6-DC- and C14-DC-ACs; Figure 2F) were also discriminated among three groups. Predictably, LCACs, OH-ACs and DC-ACs were more discriminable than other ACs species among three group.

##### 3.1.2.2 Multivariate statistical analysis of ACs in three groups of children

**FIGURE 3:**
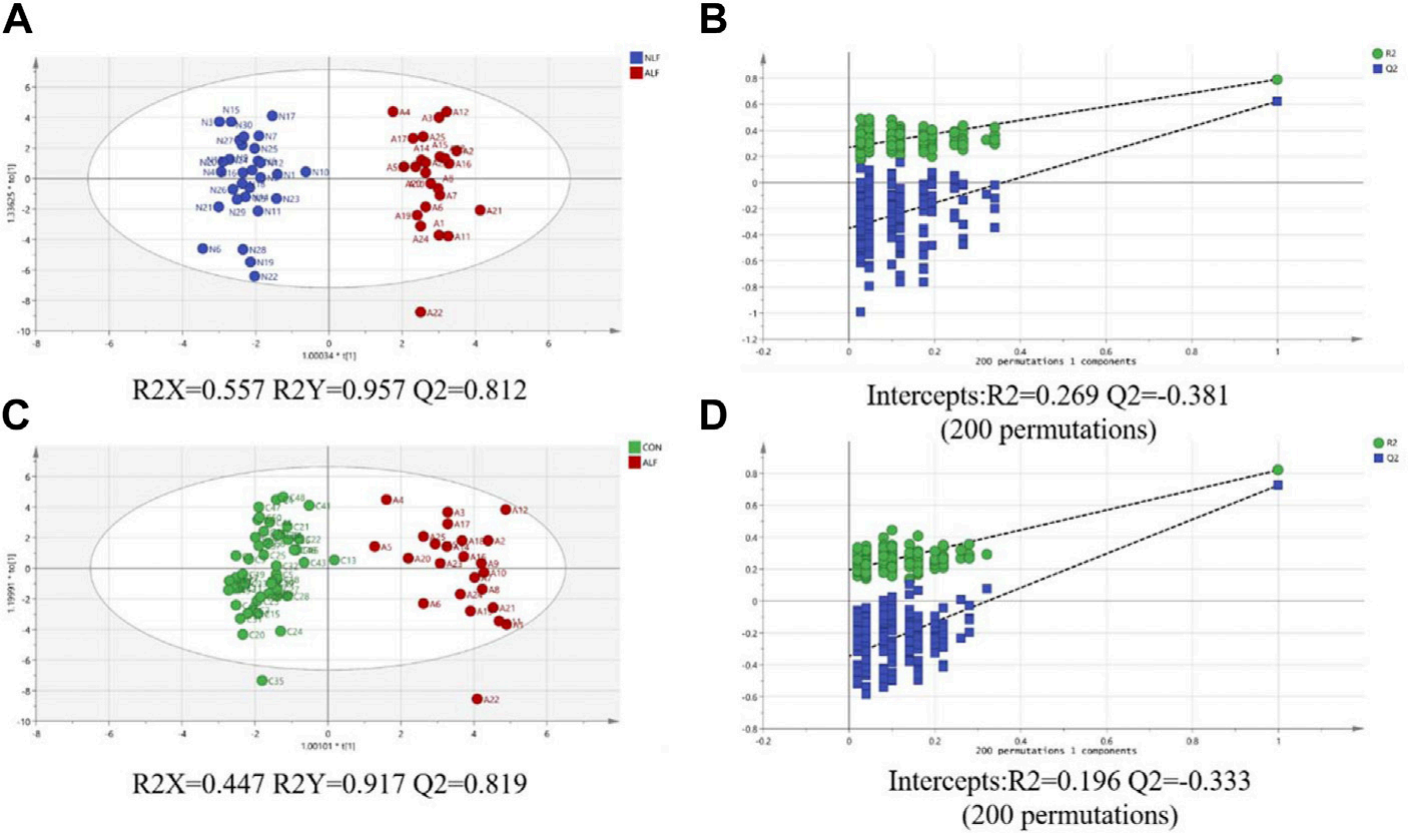
**(A)** Score plot of the OPLS-DA model between ALF and NLF groups; **(B)** Cross-validation plot of OPLS-DA mode with 200 times permutation tests between ALF and NLF groups; **(C)** core plot of the OPLS-DA model between ALF and CON groups; **(D)** Cross-validation plot of OPLS-DA mode with 200 times permutation tests between ALF and CON groups.

An OPLS-DA model was performed after UV scaling to obtain the maximum separation between ALF group and two other groups. As shown in Figure 3 A and C, the sample distribution pattern in the scores plot showed both control group and NLF group have a clear separation from ALF group, indicating that the ACs profile of the ALF children was significantly different from that of the other two groups. R2Y in OPLS-DA model is 0.957, 0.917, respectively (>0.6), indicating that the model has good stability; the prediction rate Q2 is 0.812, 0.819, respectively (>0.5), indicating that OPLS-DA model has good predictability. Permutation test (*n* = 200) on the OPLS-DA model were performed to further investigate whether there is overfitting of the model (Figures 3B,D). The results suggest that the OPLS-DA model is not overfitted.

##### 3.1.2.3 Characteristics of VPA-induced abnormal liver function ACs in children’s serum

To further investigate the metabolic profile of VPA abnormal liver function, based on the OPLS-DA model (Figure 3) between ALF and NLF groups above (with VIP >1.0), in combination with unpaired *t* test analysis (*p* < 0.05) and Fold change >1.5 (Figure 2), a total of 12 serum ACs were considered most responsible for observed differences between ALF and NLF group. Moreover, correlation analysis was performed between identified differential ACs and serum ALT (Table 1), demonstrating that increased ACs are significantly and positively correlated to the severity of abnormal liver function (*p* < 0.05).

**TABLE 1:** Identifcation of the most differential ACs between ALF and NLF groups.

Different ACs	VIP	Fold change	Correlation with ALT
C5:1	1.39929	2.425	+0.527***
C14	1.17125	1.858	+0.283**
C14:1	1.17292	1.828	+0.288**
C16	1.02006	1.564	+0.221^a^
C18	1.44373	1.660	+0.477***
C18:1	1.44215	3.326	+0.557***
C24	1.30025	2.345	+0.495***
C26	1.37469	2.305	+0.490***
C5-OH	1.1417	1.811	+0.514***
C14-OH	1.3489	3.951	+0.587***
C16-OH	1.25069	2.665	+0.576***
C6DC	1.13008	1.719	+0.351***

^a^ *p <* 0.05, **p < 0.01, ***p < 0.01correlated to ALT.

### 3.2 Expression of ACs metabolism-related genes in VPA-induced liver injury

#### 3.2.1 Liver injury in mice treated with VPA

**FIGURE 4:**
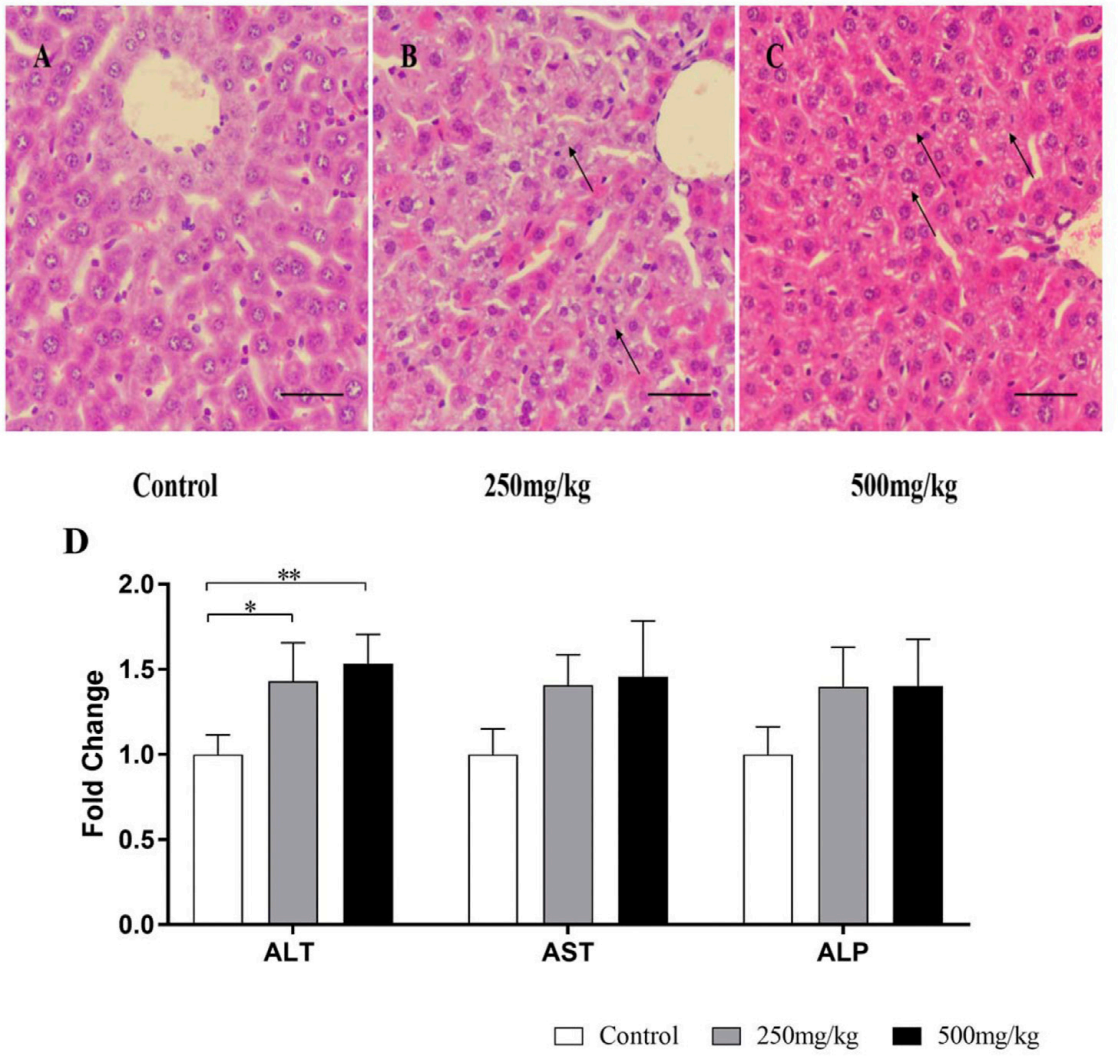
Liver injury in mice treated with VPA. Mice were treated with VPA at 250 or 500 mg/kg or the same volume of saline (5 ml/kg) once daily by intragastric administration for 28 days. **(A–C)** Hematoxylin and eosin staining of the liver sections. Representative images are shown (scale bar, 50 µm). Black arrows indicate hepatocyte microvesicular steatosis. **(D)** Effects of VPA on plasma, ALT, AST, ALP Data are expressed as the mean ± SD (*n* = 6). *p < 0.05, **p < 0.01. ALP, alkaline phosphatase; ALT, alanine transaminase; AST, aspartate transaminase.

As shown in Figure 4D, after VPA administration for 28 days, the ALT levels in plasma were significantly higher in mice treated with VPA (250 and 500 mg/kg) and plasma AST and ALP tended to increase among three groups. Histopathologically, H&E staining results revealed that VPA caused hepatic steatosis characterized by microvesicular steatosis. These results suggested that exposure to VPA induced steatosis in mouse livers.

#### 3.2.2 Liver injury in LO2 cell treated with VPA

**FIGURE 5:**
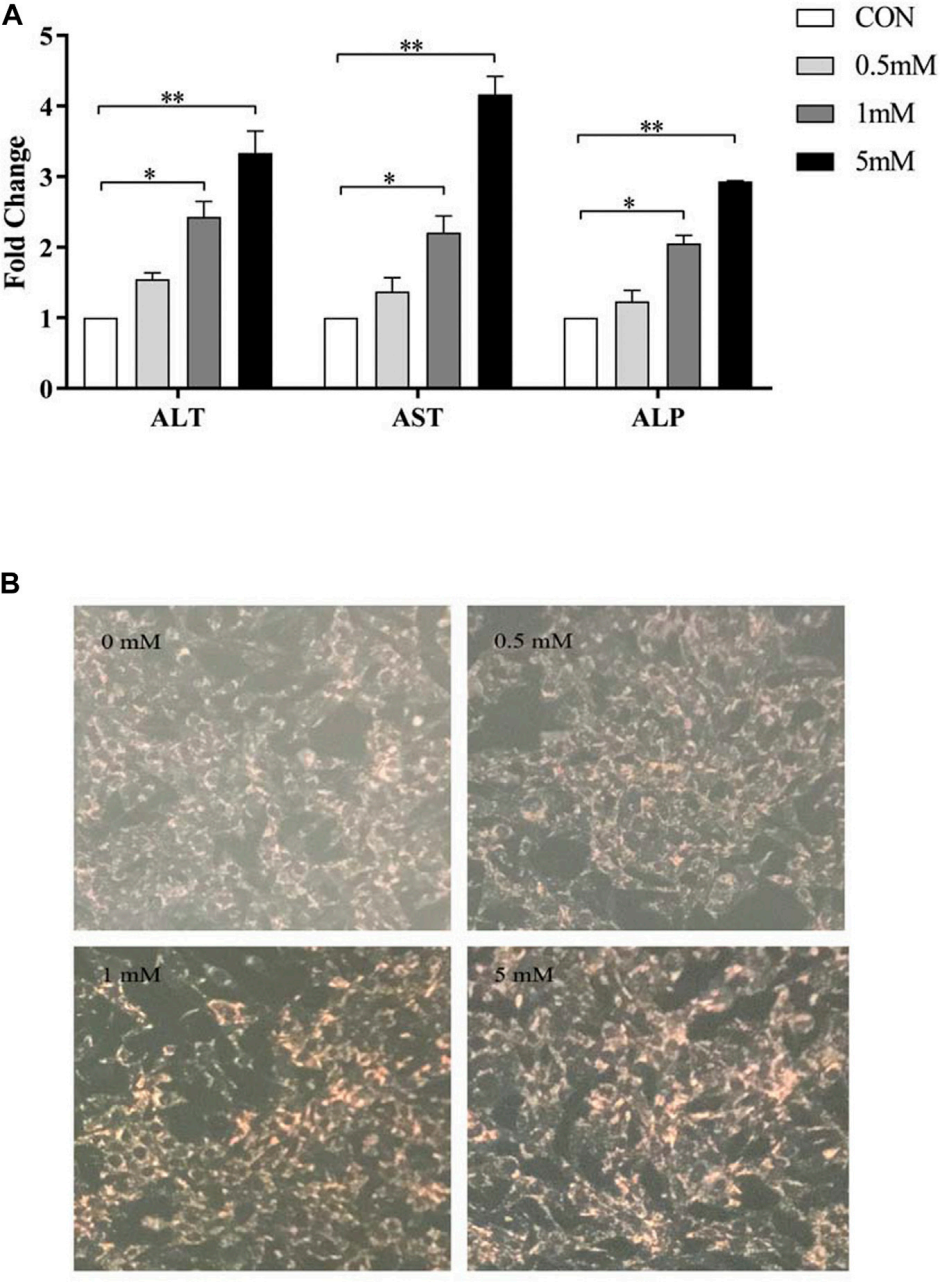
Toxic effects of different concentrations of VPA on LO2 cells. **(A)** Effects of different concentrations of VPA (0, 0.5, 1, 5 mM) on ALT, AST and ALP enzyme activities in LO2 cells; **(B)** Oil red O staining to observe the effects of different concentrations of VPA (0, 0.5, 1, 5 mM) on intracellular lipid levels in LO2 cells, data are expressed as mean ± SEM (*n* = 3). *p < 0.05, **p < 0.01.

ALT, AST and ALP enzyme activities in LO2 cells treated with different concentrations of VPA (0, 0.5, 1 and 5 mM) for 24 h were significantly increased (*p* < 0.05; Figure 5). Since the pathological manifestation of VPA-induced hepatotoxicity was microvesicular steatosis, oil red O staining was performed on LO2 cells treated with different concentrations of VPA. The results are shown in Figure 5 B. Only a small amount of red lipid droplets were found in the control and 0.5 mM VPA-treated cells, while the LO2 cells treated with 1 mM and 5 mM VPA the intracellular red lipid droplets were significantly increase and the cell morphology was changed. The above results indicate that VPA has toxic effects on LO2 cells and can lead to intracellular lipid accumulation.

#### 3.2.3 Expression of genes and proteins related to ACs in mice and cell

**FIGURE 6:**
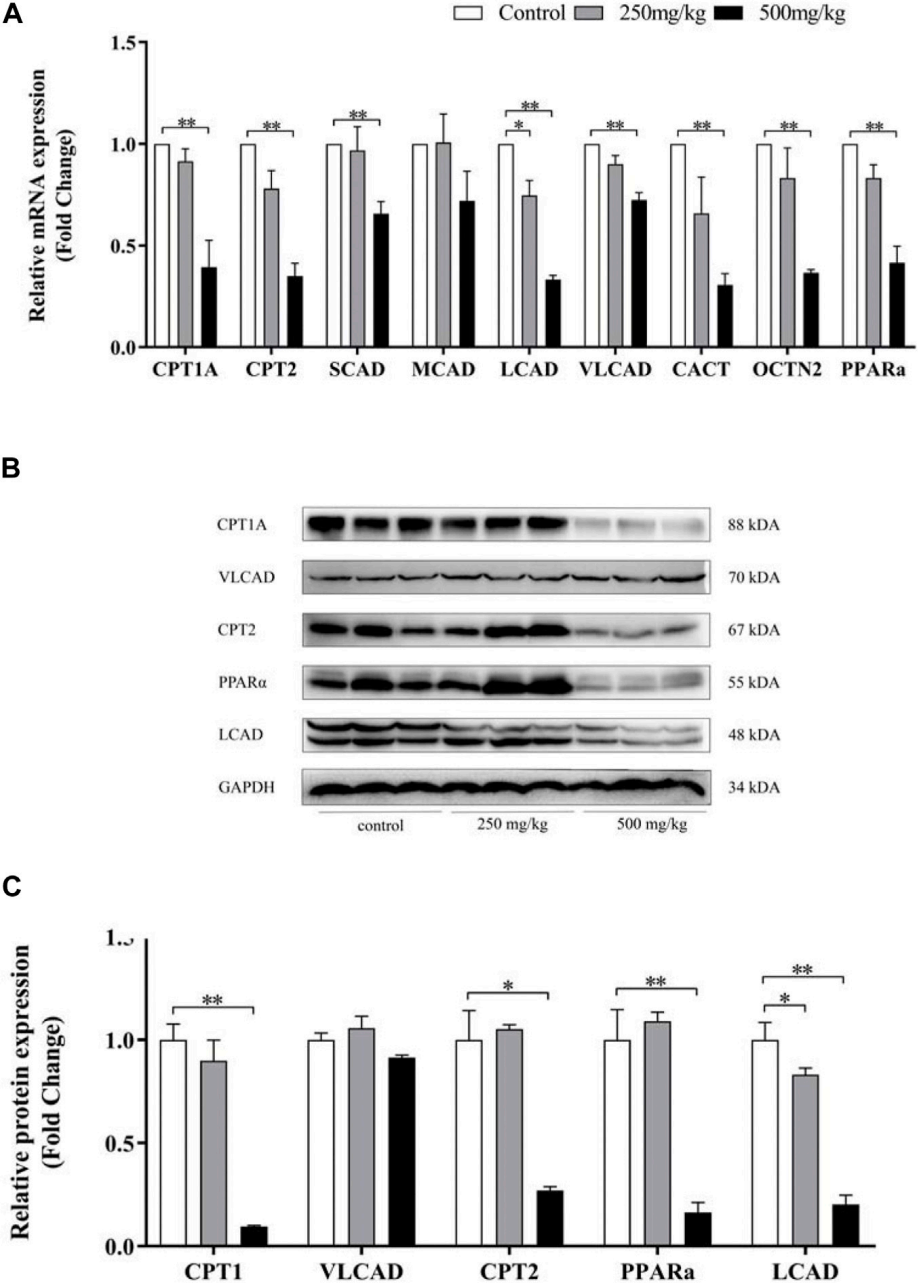
Effect of VPA on ACs-related gene expression in mouse livers. Real-time PCR analysis of the genes **(A)** and western blot analysis **(B–C)** of proteins involved in ACs metabolism. Data are expressed as the mean ± SD (*n* = 6). *p < 0.05, **p < 0.01.

ACs accumulation (especially LCACs) in VPA-induced abnormal liver function was further investigated by analysing the mRNA and protein levels of genes involved in ACs metabolism by real-time PCR and western blotting, respectively (Figure 6 A–C). Levels of Pparα, Cpt1a and Cpt2 were significantly decreased in mice in the 500 mg/kg group than in the other groups. Decreased levels of Lcad, but not Mcad, were also observed. In mice in the 250 mg/kg, only levels of Lcad was decreased compared with control group. Consistent with the mRNA expression results, mice in the 500 mg/kg group demonstrated decreased protein expression of Lcad.

**FIGURE 7:**
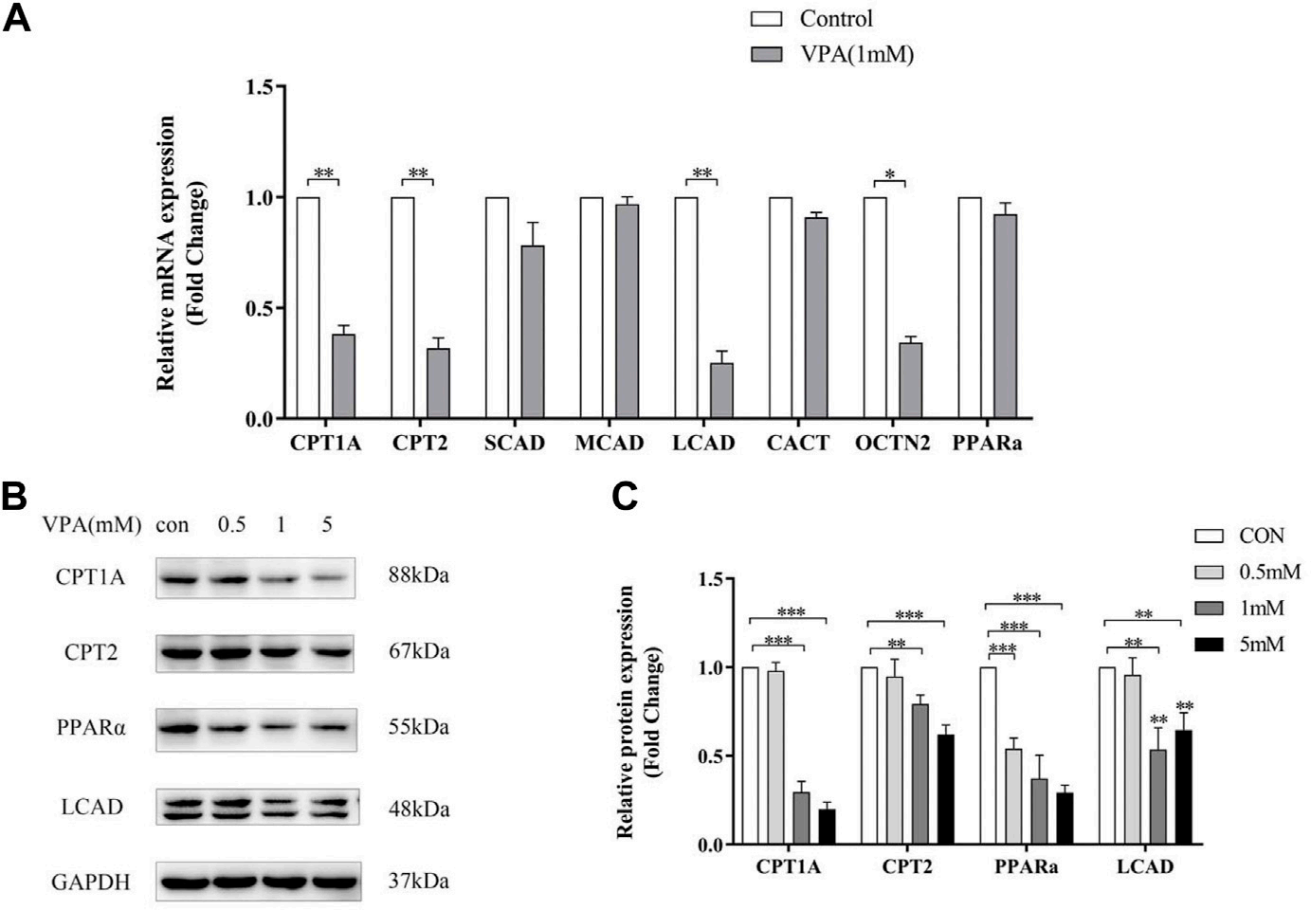
Effect of VPA on ACs-related gene expression in LO2 cell. **(A)** Real-time PCR analysis of VPA (1 mM) on ACs-related metabolism genes. **(B–C)** Western blot analysis of the effect of VPA on protein expression of ACs -related genes. Data are expressed as the mean ± SD (*n* = 3). *p < 0.05, **p < 0.01, ***p < 0.01.

Quantitative PCR and Western Blotting were used to examine the expression of acylcarnitine-related genes and proteins in LO2 cells after 24h treatment with VPA. Similar to the results in animal models, VPA significantly down-regulated Cpt1a, Cpt2 and Lcad mRNA and protein expression, and the down-regulation was proportional to the dose of VPA (Figure 7 A–C). In LO2 cells, 1 mM VPA had no effect on the mRNA expression of Scad and Mcad (*p* > 0.05, Figure 7 A), which may explain the absence of changes in SCACs and MCACs in the ALF group compared with the NLF group. The above results suggest that VPA down-regulates the expression including Cpt1a, Cpt2 and Lcad, which may cause the accumulation of LCACs. Consistent with the results in animal models, VPA down-regulated Pparα expression (*p* < 0.05).

### 3.3 PPARα activation by feno protected against VPA-induced abnormal liver function

#### 3.3.1 Effect of agonistic PPARα on VPA-induced hepatotoxic mice

**FIGURE 8:**
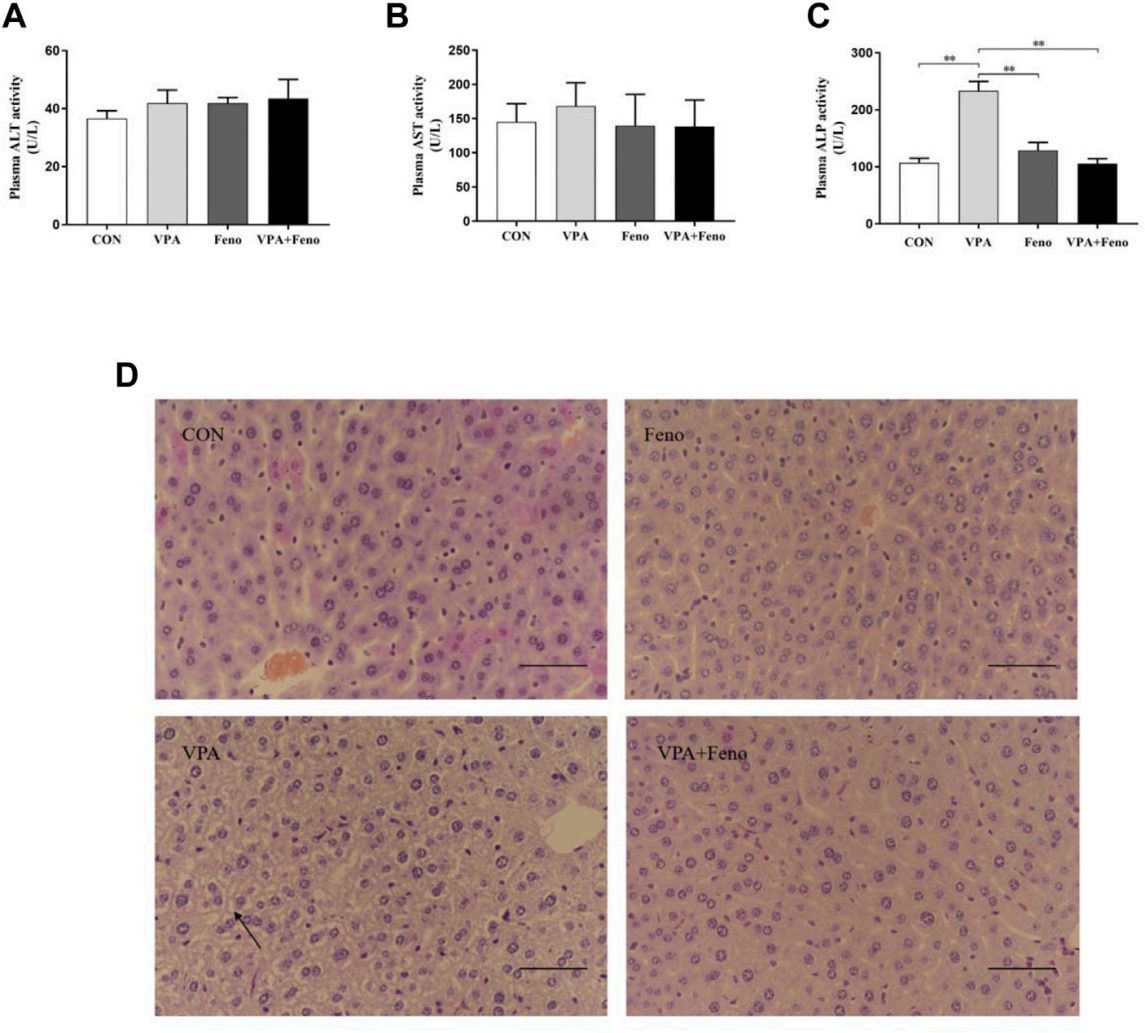
Effect of Feno on liver tissue morphology and biochemical indexes of liver function in VPA-induced hepatotoxic mice. **(A–C)** Changes of Feno on biochemical indexes of plasma liver function in mice **(D)** H and E staining to observe the effect of Feno on liver tissue structure in mice (scale bar, 50 μm); black arrow: microvesicular steatosis. Data are expressed as mean ± SD (*n* = 6). *p < 0.05, **p < 0.01; CON: control group; VPA: VPA 500 mg/kg group; Feno: Feno50 mg/kg group; VPA + Feno: VPA 500 mg/kg + Feno50 mg/kg group; ALP, alkaline phosphatase; ALT, alanine aminotransferase; AST, aspartate aminotransferase.

As shown in Figure 8 A-C, ALT and AST in plasma also tended to increase in VPA-treated mice, and ALT and AST levels decreased after co-administration of Feno. As shown in Figure 8 C, ALP levels in plasma were significantly elevated in VPA-treated mice (*p* < 0.05), and normalized after co-administration of Feno. As shown in Figure 8 D, the H&E staining results showed that microvesicular steatosis (shown by black arrows) could be significantly observed in the liver of mice in the VPA group, and the structure of liver lobules was destroyed, while after Feno was combined, the structure of liver lobules was intact and only a small portion of microvesicular steatosis appeared. These results suggest that Feno can ameliorate VPA-induced hepatic steatosis in mice.

#### 3.3.2 Effect of agonistic PPARα on lipid accumulation in VPA-induced hepatotoxic mice

##### 3.3.2.1 Data quality assessment

**FIGURE 9:**
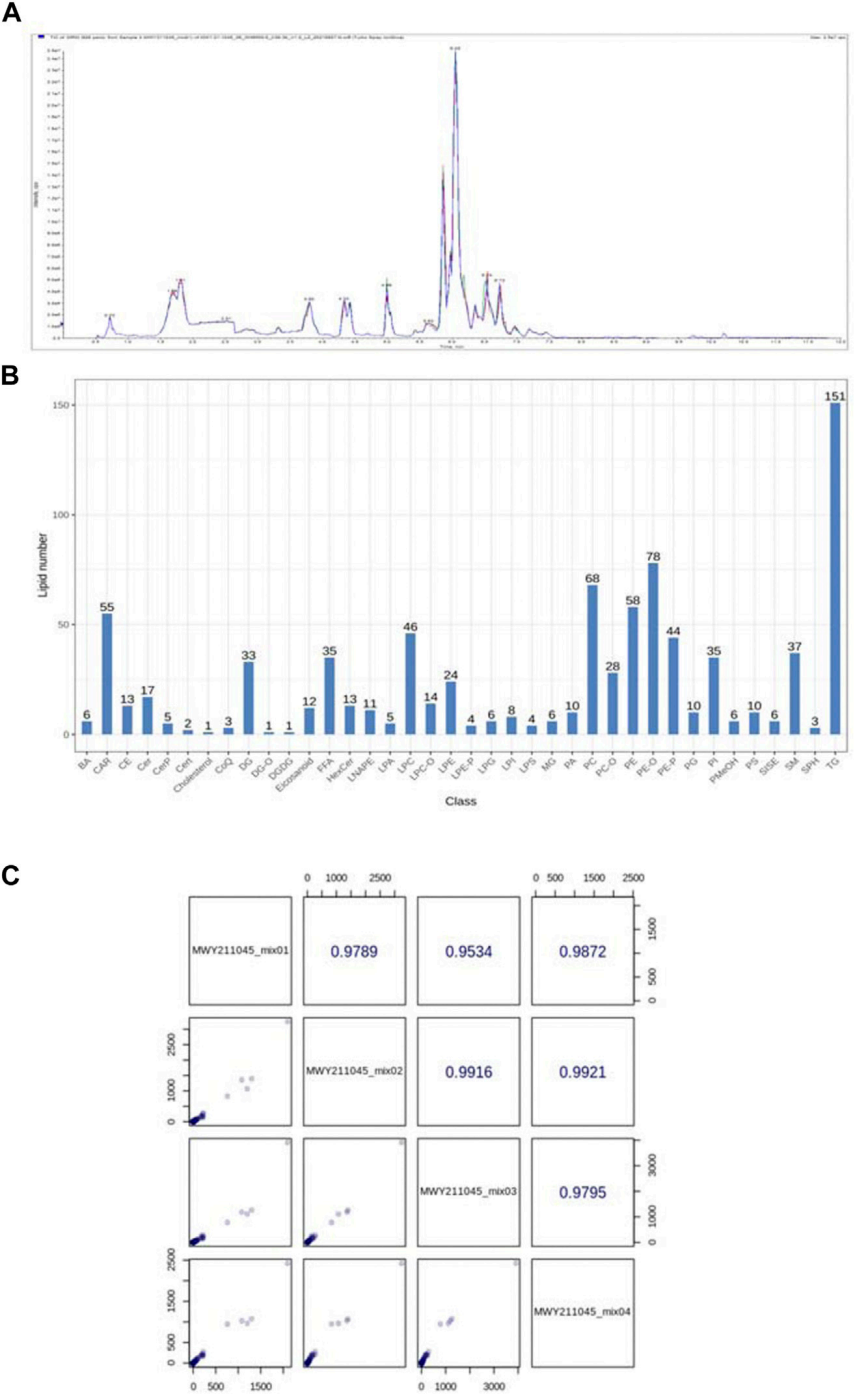
**(A)** Total ion current (TIC) of mixed samples by mass spectrometry **(B)** Lipid species and distribution identified by quantitative plasma lipidomics; **(C)** Pearson correlation analysis of QC samples.

As shown in Figure 9, for the alteration of plasma lipid composition by Feno on VPA-induced hepatotoxic mice, this study utilized quantitative lipidomics based on the UPLC-(±) ESI-TOF/MS technique to analyze the changes of each lipid component in mouse plasma. The mixed solution was used as QC samples, and a QC sample was inserted every ten detection analysis samples during the instrument analysis. The excellent stability of the instrument could be judged by overlapping display analysis of the total ion flow chromatogram (TIC) of the mass spectrometry detection analysis of the same QC sample (Figure 9A). Pearson correlation analysis was performed on the QC samples, and the higher correlation of QC samples (|r | closer to 1) indicates the better stability of the whole detection process and the higher data quality. The strength of the instrument can be judged as good from the figure (Figure 9C). Based on the precise m/z, retention time, and MS/MS, a total of 869 lipid components were identified using the established lipidomics technique in positive and negative ion mode, covering eight different lipid subclasses (Figure 9 B), of which the maximum number of TG species identified was 151.

##### 3.3.2.2 Lipid composition analysis

The composition of lipids is sample-specific, and different types of samples contain different lipid classes and ratios; in addition, lipid composition can change during different treatments or biological processes. The analysis of lipid composition ratios can examine the distribution of major lipids in the samples as a whole. Lipid subclass composition ring diagram for each group of samples were shown in Supplementary Figure S1.

##### 3.3.2.3 Changes in lipid subclass content

**FIGURE 10:**
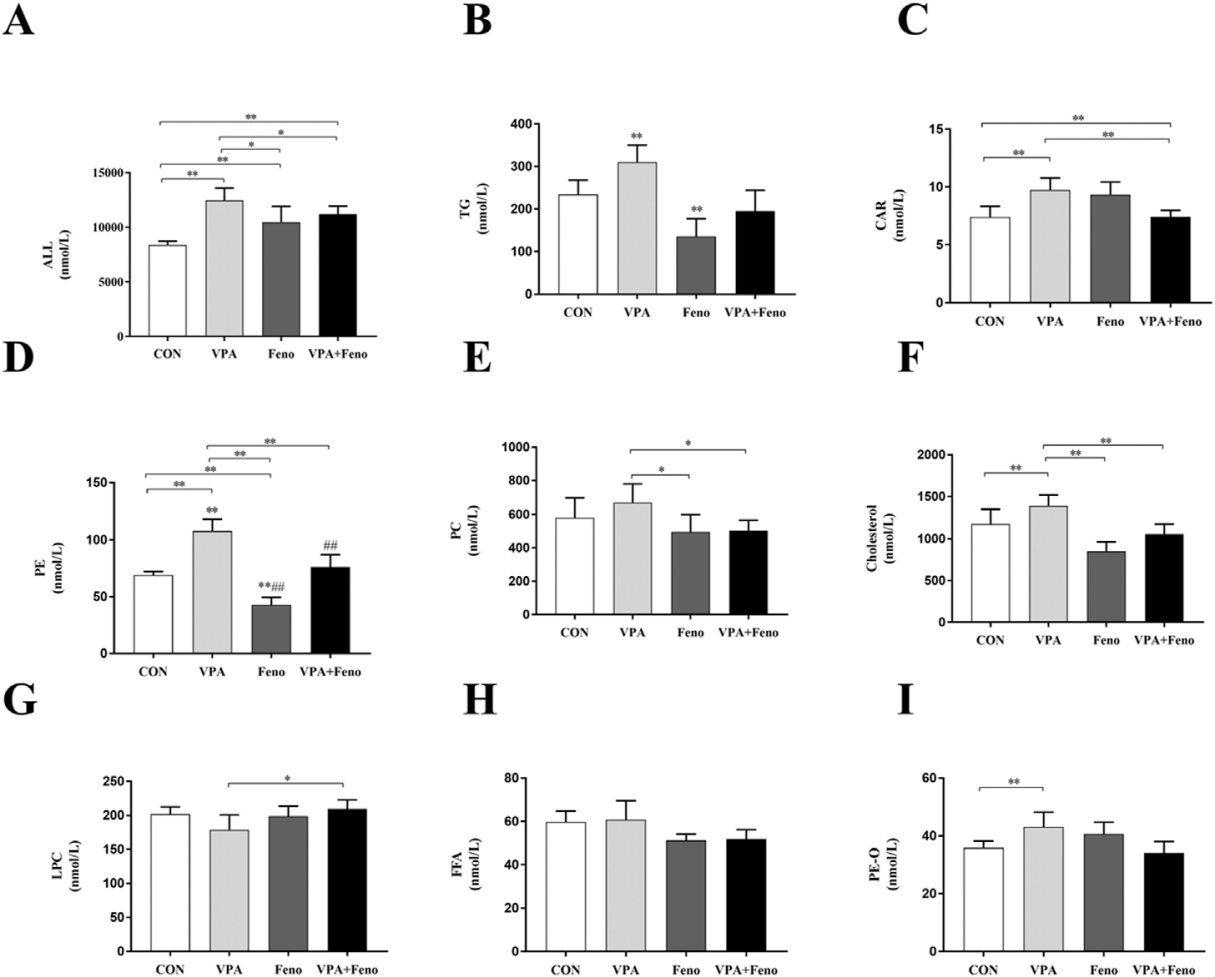
Changes in total lipid and subclass content between four groups. **(A)** Total lipid content; **(B)** TG **(C)** CAR **(D)** PE **(E)** PC **(F)** Cholesterol **(G)** LPC **(H)** FFA **(I)** PE-O levels; CON: control group; VPA: VPA 500 mg/kg group; Feno: Feno50 mg/kg group; VPA + Feno: VPA 500 mg/kg + Feno50 mg/kg group; *p < 0.05, **p < 0.01. TG: triglycerides, CAR: acylcarnitine, PE: phosphatidylethanolamine, PC: phosphatidylcholine, LPC: lysophosph-atidylcholine, FFA: free fatty acids.

To investigate the changes in plasma lipid composition of Feno on VPA -induced hepatotoxic mice, the total amount of lipid molecules and the components of the eight lipid subclasses with higher content were compared between groups (shown as Figure 10). Compared with the control group, the levels of total lipid molecules, TG, CAR, PE, PC, Cholesterol, and PE-O were significantly increased in the VPA group (*p* < 0.05), and the levels of total lipid molecules, TG, CAR, PE, PC, and Cholesterol were significantly decreased after Feno was combined with the VPA group (*p* < 0.05), demonstrating that Feno was able to significantly improve the lipid accumulation caused by VPA.

##### 3.3.2.4 Alteration of plasma lipid composition in VPA-induced hepatotoxic mice by agonistic PPARα

**FIGURE 11:**
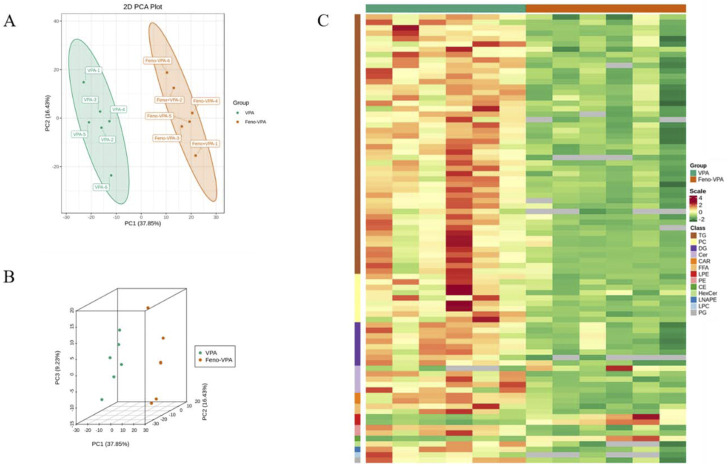
Alteration of plasma lipid composition in VPA-induced hepatotoxic mice by Feno. **(A–B)** Principal component analysis between the two groups **(C)** Heat map of differential metabolite clustering between the two groups. Vpa: VPA 500 mg/kg group; Feno + VPA: Feno50 mg/kg + VPA 500 mg/kg group.

As shown in Figure 11 A-B, principal component analysis was performed between the VPA group and the Feno + VPA group, and it was found that the samples were better differentiated between the two groups, which proved that the lipid composition was more altered between the two groups. The clustering heat map of differential metabolites between the two groups was plotted, and Scale was the expression obtained after standardization (the redder the color, the higher the expression), and it could be seen that the content of all kinds of lipid components decreased after the combination of Feno, among which the most obvious difference was the TG class lipids, which proved that Feno could reduce the lipid accumulation in the plasma of VPA-induced hepatotoxic mice.

##### 3.3.2.5 Effect of Feno on plasma ACs profiles in VPA-induced hepatotoxic mice

**FIGURE 12:**
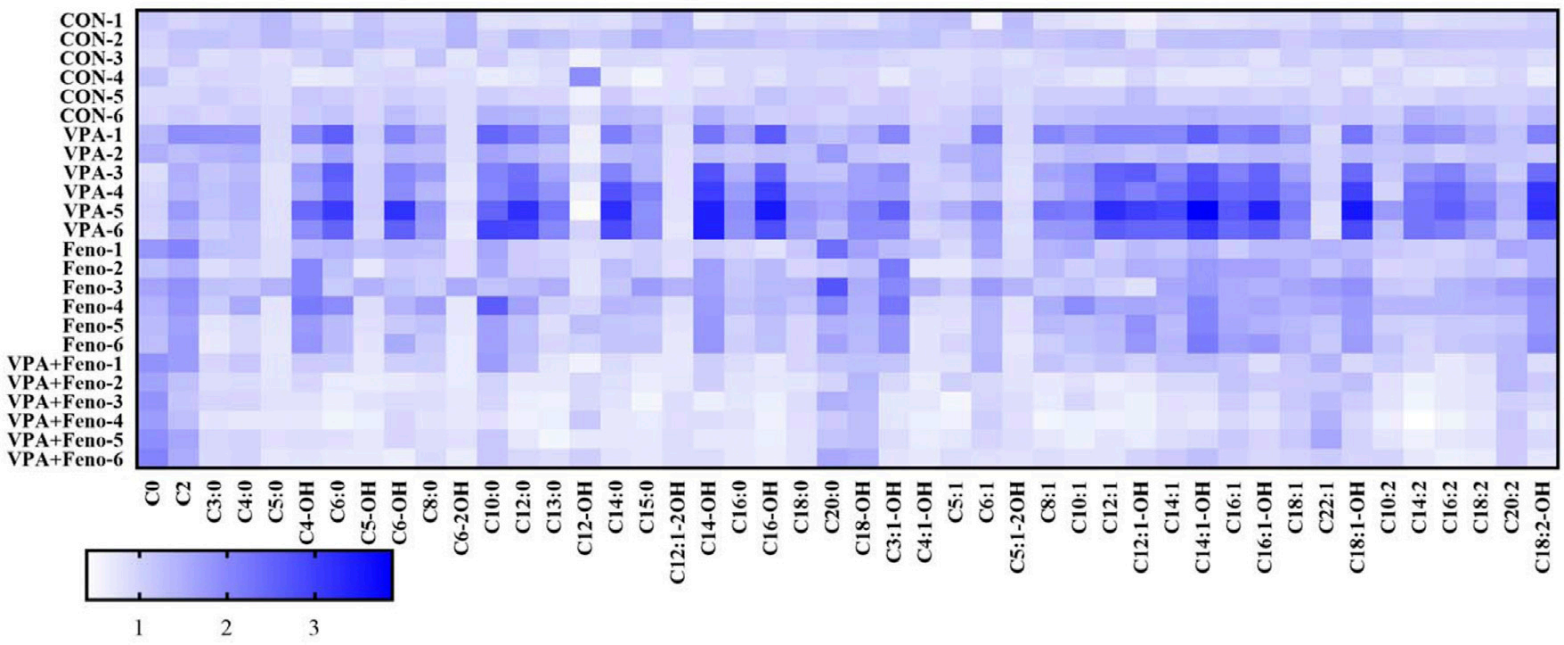
Heat map analysis of the effect of Feno on plasma ACs profile in VPA-induced hepatotoxic mice. CON: control group; VPA: VPA 500 mg/kg group; Feno: Feno50 mg/kg group; VPA + Feno: VPA 500 mg/kg + Feno50 mg/kg group.

To visually obtain the overall differences in ACs profiles between the control, VPA and Feno and VPA + Feno groups, changes in ACs profiles between groups were analyzed using heat maps (Figure 12), and it was found that plasma ACs concentrations were significantly increased in VPA-induced hepatotoxic mice compared with the control group (blue part of the figure), with the most significant increase in LCACs including C14, C14-OH, C14:1, consistent with the differential ACs composition screened within human serum. After the combination of Feno, ACs levels were significantly decreased compared to the VPA group (white part), demonstrating elevated levels of mitochondrial fatty acid β-oxidation.

#### 3.3.3 Impaired mitochondrial β-FAO was recovered by feno

**FIGURE 13:**
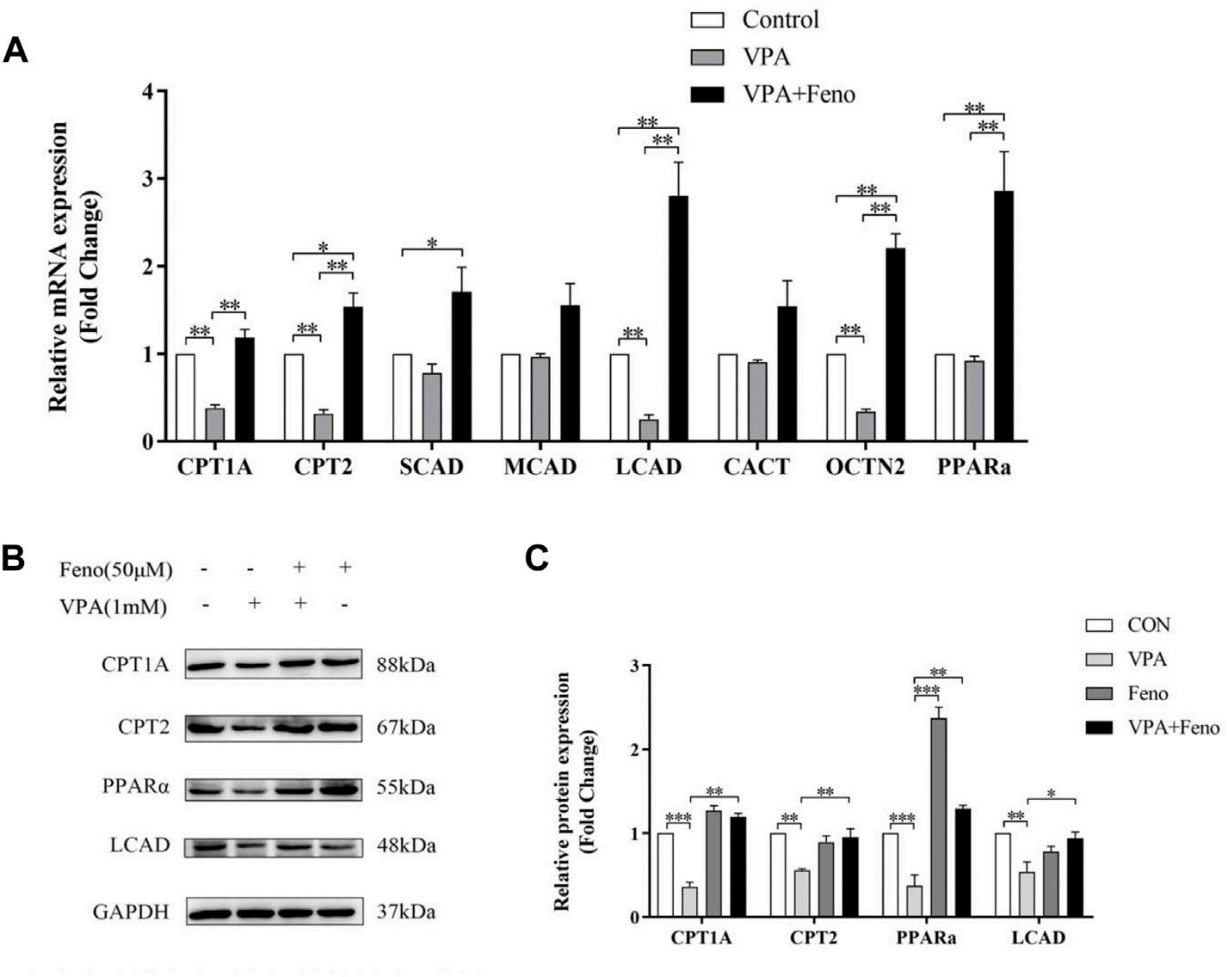
Effect of Feno on the expression of ACs-related genes. **(A)** Real-time PCR technique to detect the effect of VPA (1 mM) and Feno (50 μM) on ACs-metabolism-related genes. **(B–C)** Western blot detection of the effect of VPA and Feno (50 μM) on protein expression of ACs-metabolism-related genes. Data are expressed by mean ± SD (*n* = 3). *p < 0.05 **p < 0.01.

CPT1A, CPT2 and LCAD were the mitochondrial β-FAO relevant genes regulated by PPARα, so they could be activated by Feno. VPA was found to significantly inhibit the protein expression level of PPARα (Figure 13C), Quantitative real-time PCR (qPCR) and western blot analysis indicated Feno could enhance the expression of genes for CPT1A, CPT2 and LCAD in LO2 cells and reverse the inhibitory effect of VPA on β-FAO (Figures 13A–C).

#### 3.3.4 Oxidative stress in VPA-induced abnormal liver function was eliminated by feno

**FIGURE 14:**
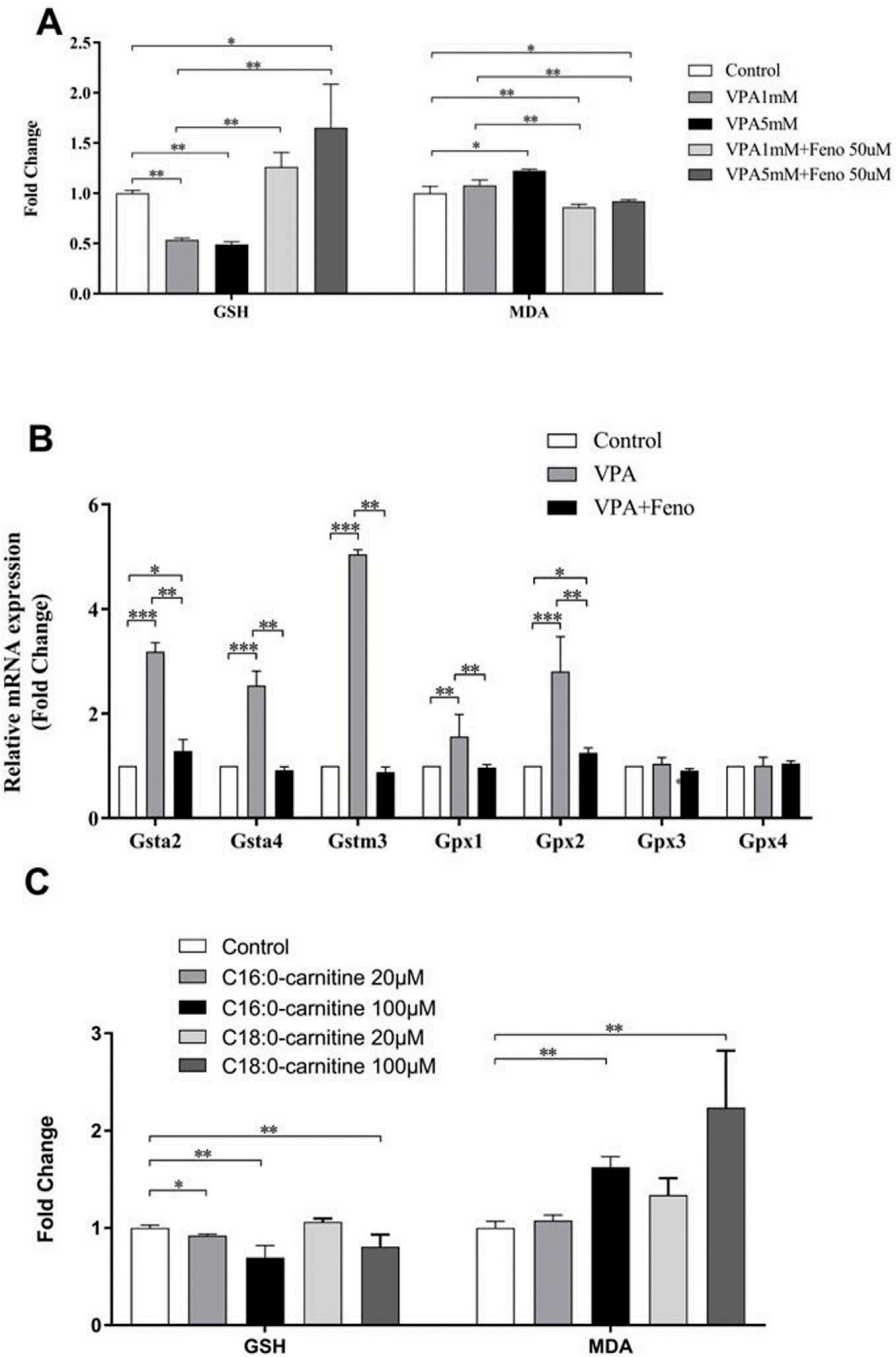
Effect of Feno on oxidative stress caused by VPA. **(A)** Effects of VPA on MDA and GSH levels and the reversal of Feno; **(B)** Effects of VPA on several antioxidant genes and the reversal of Feno; **(C)** Effects of LCACs (C16:0-carnitine and C18:0-carnitine) on MDA and GSH levels. Data are expressed by mean ± SD (*n* = 3). *p < 0.05 **p < 0.01.

The results of the present study showed that VPA could increase oxidative stress levels *in vivo*, with decreased levels of malondialdehyde (MDA), an indicator of oxidative stress, and increased levels of glutathione (GSH) (Figure 14A). After exposure to palmitoylcarnitine (C16:0-carnitine) and octadecanoylcarnitine (C18:0-carnitine) for 24 h, LO2 cells showed an increase in MDA, an indicator of oxidative stress, and a decrease in GSH, demonstrating that the accumulation of long-chain ACs may be one of the reasons for oxidative stress caused by VPA (Figure 14C). It can be seen that the expression levels of several antioxidant genes were up-regulated in the VPA group (Figure 14B), including glutathione S-transferases (Glutathione S transferase alpha (GSTA)2, GSTA4, and Glutathione S transferase M3 (GSTM3)) and glutathione peroxidase (Glutathione Peroxidase (GPX)1, GPX2). Addition of Feno significantly decreased the levels of the antioxidant genes GSTA 2, GSTA 4, GSTM3, GPX1, and GPX2 (Figure 14B, *p* < 0.01). The levels of MDA and GSH in the liver also returned to normal levels (*p* < 0.01) (Figure 14A).

## 4 Discussion

### 4.1 ACs as biomarkers of mitochondrial dysfunction in VPA-induced abnormal liver function

Serum ACs profiling is a well-established clinical test and is currently used to screen for inborn metabolic disorders such as mitochondrial fatty acid oxidative deficiency in newborns and is an essential biomarker of mitochondrial dysfunction. VPA, a branched-chain fatty acid, is involved in fatty acid β-oxidation and can lead to alterations in the carnitine profile. It has been demonstrated (Nakajima et al., 2011)that VPA can affect free carnitine and ACs homeostasis. Still, these studies focused on the alteration of serum ACs composition in patients treated with long-term VPA without focusing on the association between VPA-induced hepatotoxicity and ACs composition. The serum ACs/free carnitine ratio is an important indicator of ACs homeostasis *in vivo*, and consistent with the results of previous studies (Silva et al., 2001b), the present study found an increase in the ACs/free carnitine ratio in the two groups of children taking VPA compared to the control group, with a 1.36- and 3.46-fold increase, respectively.

Unlike previous studies, the present study focused for the first time on changes in serum ACs profiles in children with epilepsy with abnormal liver function caused by VPA. ACs was increased in the ALF group compared with both the control and NLF groups, which was statistically significant (*p <* 0.01), and there was no significant difference in ACs concentration in the NLF group compared with the control group (*p >* 0.05). The OPLS-DA model was used to analyze further the differences in ACs components between the ALF and NLF groups. Finally, 12 different ACs components were screened proved that these specific ACs components might be related to VPA-induced hepatotoxicity.

The accumulation of multiple ACs (especially LCACs) in children with abnormal liver function caused by VPA indicates dysfunction of mitochondrial fatty acid β-oxidation. This dysfunction may be caused by the inhibition of multiple enzymes and/or transporter proteins involved in fatty acid β-oxidation metabolism by VPA (Rinaldo et al., 2008), i.e., inhibition of numerous enzymes involved in catalyzing the conversion of acyl-coenzyme A to ACs, including CPT1, CPT2, Carnitine acyl transferase (CACT), LCAD, and VLCAD (Violante et al., 2013); and OCTN2(Organic Cation/Carnitine Transporter 2), a protein involved in cellular acylcarnitine transport (Mccoin et al., 2015). In addition, the increase in DC-ACs, OH-ACs, and VLCACs (e.g., C26-AC) reflects the disruption of fatty acid metabolism outside the mitochondria, primarily associated with impaired metabolism of the peroxisome as well as the endoplasmic reticulum (Wanders et al., 2016). VPA inhibits fatty acid β-oxidation, and the accumulation of large amounts of fat in hepatocytes triggers lipid peroxidation, which ultimately leads to liver injury (Iman et al., 2013).

Studies on the inhibitory effect of VPA on mitochondrial fatty acid β-oxidation have shown that VPA has different inhibitory effects on fatty acid oxidation of different chain lengths. Silva et al. (Silva et al., 2001a)showed that in rat hepatocytes, the inhibitory effect of VPA on fatty acid β-oxidation was more pronounced for long-chain fatty acids than for medium-chain fatty acids; VPA induced the accumulation of LCACs in fibroblasts. Consistent with the above findings, the down-regulation of *CPT1A, CPT2,* and *LCAD* gene and protein expression by VPA was found to be significantly greater than that of *SCAD* and *MCAD* in the present study. VPA was found to down-regulate the level of *SCAD* mRNA only in the liver of mice at 500 mg/kg (*p <* 0.05). This finding explains why the accumulation of LCACs occurred in the ALF group compared with the NLF group, while the levels of *SCACs* and *MCACs* were unchanged. In addition, we found that the downregulation of ACs metabolism-related genes and protein expression by VPA in both mouse liver and hepatocytes was dose-dependent, i.e., the greater the VPA concentration, the more pronounced the downregulation effect.

Carnitine inborn metabolic disorders (IEM) can be classified as disorders of carnitine biosynthesis, carnitine transport disorders, and mitochondrial carnitine-acyl carnitine recycling. Human mitochondrial carnitine-acyl carnitine cycle disorders cause inherited disorders such as CACT, CPT2, and LCAD deficiencies, resulting in impaired fatty acid β-oxidation and elevated serum ACs levels. CACT deficiency is a rare autosomal recessive disorder caused by SLC25A20 deficiency (Indiveri et al., 2011), diagnosed as ACs (especially C16- and C18:1-AC) and reduced levels of free carnitine (Rubio-Gozalbo et al., 2004). The diagnosis of CPT2 deficiency is based on elevated levels of C12- to C18-ACs, specifically increased levels of C16- and C18:1-ACs and reduced levels of free carnitine. (C16 + C18:1)/C2 is an important indicator for detecting CPT2 deficiency (Tajima et al., 2017). Similarly, LCAD deficiency leads to impaired fatty acid β-oxidation, which also causes metabolic disturbances in ACs (van Vlies et al., 2005). In the present study, similar to IEM described above, VPA downregulated the expression of *CACT, CPT2*, and *LCAD*, partially explaining the accumulation of ACs in children with abnormal liver function caused by VPA.

Carnitine supplementation in IEM patients is a more routine tool but is indeed controversial. There is concern that the large accumulation of ACs may be toxic in long-chain fatty acid oxidation disorders (Almannai et al., 2019). In a case report, two patients with VLCAD deficiency developed more frequent rhabdomyolysis after carnitine supplementation (Watanabe et al., 2018). In a mouse model, LCACs accumulated in mitochondria of the ischemic heart inhibited oxidative phosphorylation (Watanabe et al., 2018). Another study found that LCACs modulate genes encoding the alpha subunit of the potassium channel and promote the development of arrhythmias (Ferro et al., 2012). Based on this potential risk, carnitine therapy should be avoided during acute metabolic disorders (Spiekerkoetter et al., 2009). Although carnitine supplementation has been widely used to prevent and treat fatty liver toxicity caused by VPA, recent studies have shown that carnitine supplementation has a minimal therapeutic effect in patients with acute toxicity caused by VPA(Nguyen et al., 2022). On the other hand, since carnitine supplementation may increase ACs accumulation, the search for a potential protective agent against VPA-induced hepatotoxicity that both upregulates fatty acid β-oxidation in mitochondria and does not cause ACs accumulation is necessary.

### 4.2 Protective role of feno by regulation of ACs-related gene expression in VPA-induced abnormal liver function

Peroxisome proliferator-activated receptors (PPARs) are involved in various lipid and glucose metabolic pathways (Pawlak et al., 2015). It has been suggested that activation of PPARα may protect against hepatic steatosis (Beyoğlu and Idle, 2013; Yang et al., 2016; Chascsa et al., 2017) Feno effectively improved the atherogenic lipid profile associated with T2DM by activating PPARα(Rosenson, 2009). Experimental evidence suggests that Feno has multiple protective effects against hepatic steatosis (Li et al., 2012; El-Sisi et al., 2013; Chuanyong et al., 2015; García- Ca?averas et al., 2016). Activation of PPARα mediated with Feno may enhance the expression of genes that promote hepatic β-oxidation. In addition, Feno reduces insulin resistance in the liver (Buldak et al., 2012) It also inhibits the expression of inflammatory mediators involved in the pathogenesis of nonalcoholic steatohepatitis (Koh et al., 2011). Feno can limit the infiltration of hepatic macrophages (Zeng et al., 2013) Other hepatoprotective effects include reduction of oxidative stress and improvement of hepatic microvascular function (Zeng et al., 2013) Experimental studies have shown that Feno can limit hepatic steatosis associated with the high-fat diet, T2DM, and obesity-related insulin resistance (Zeng et al., 2013).

The effect of Feno on various types of lipids accumulation in VPA-induced hepatotoxic mice was examined by quantitative lipidomics techniques. In this study, we found that the combination of Feno significantly reduced the total amount of lipid molecules in the plasma of VPA-induced hepatotoxic mice compared to VPA group (*p <* 0.05). The levels of various lipid subclasses such as TG, CAR, PE, PC, and Cholesterol were also significantly reduced compared to the VPA group (*p <* 0.05). Pathological examination revealed that Feno significantly improved lipid accumulation in the liver of VPA-induced hepatotoxic mice and LO2 cells. Feno significantly reduced the plasma ACs levels, especially LCACs, in VPA-induced hepatotoxic mice and restored the ACs levels to normal, demonstrating that it could facilitate the hepatotoxic effects of VPA by activating the fatty acid β-oxidation function.

We found that Feno significantly increased the expression of PPARα and its downstream target genes, including *CPT1A, CPT2, and LCAD,* in addition to increased MDA levels in VPA-induced hepatotoxic LO2 cells, indicating the activation of oxidative stress. VPA significantly increased the expression levels of antioxidant genes *GSTA2, GSTA4, GSTM3*, and *GPX2*. These findings suggest that oxidative stress may be associated with involvement in VPA-induced hepatotoxicity. *In vitro* experiments have shown that an increase in LCACs (C16 and C18) can directly increase MDA levels leading to oxidative stress. The expression of *GSTA2, GSTA4, GSTM3*, and *GPX2* levels was down-regulated. MDA and GSH returned to normal levels after co-administration of Feno, demonstrating that Feno can inhibit the onset of oxidative stress and thus reduce the toxic effects of VPA. This evidence suggests that the Feno-mediated activation of PPARα can protect against VPA-induced hepatotoxicity by regulating fatty acid β-oxidation.

In conclusion, This present study demonstrated that VPA inhibit the gene and protein expression of β-FAO enzymes *via* PPARα signaling, resulting in suppression of β-FAO, oxidative stress, and specific ACs accumulation. It is likely that PPARα activation ameliorates VPA-induced hepatotoxicity through its regulation of β-FAO genes, including *CPT1A, CPT2 and LCAD.* Activation of PPARα reduces VPA-induced lipid and ACs accumulation in multiple components and ameliorates fatty liver injury. These findings support that PPARα agonists may be potential protectors against VPA-induced hepatotoxicity.

## Data Availability

The original contributions presented in the study are included in the article/Supplementary Material, further inquiries can be directed to the corresponding author.
