# Supplementary material for: The serum acylcarnitines profile in epileptic children treated with valproic acid and the protective roles of peroxisome proliferator-activated receptor a activation in valproic acid-induced liver injury
Source: Front Pharmacol. 2022 Nov 8;13:1048728. doi: 10.3389/fphar.2022.1048728 (PMC9681037; doi:10.3389/fphar.2022.1048728)
Supplement: Supplementary file 1 [file DataSheet1.pdf]

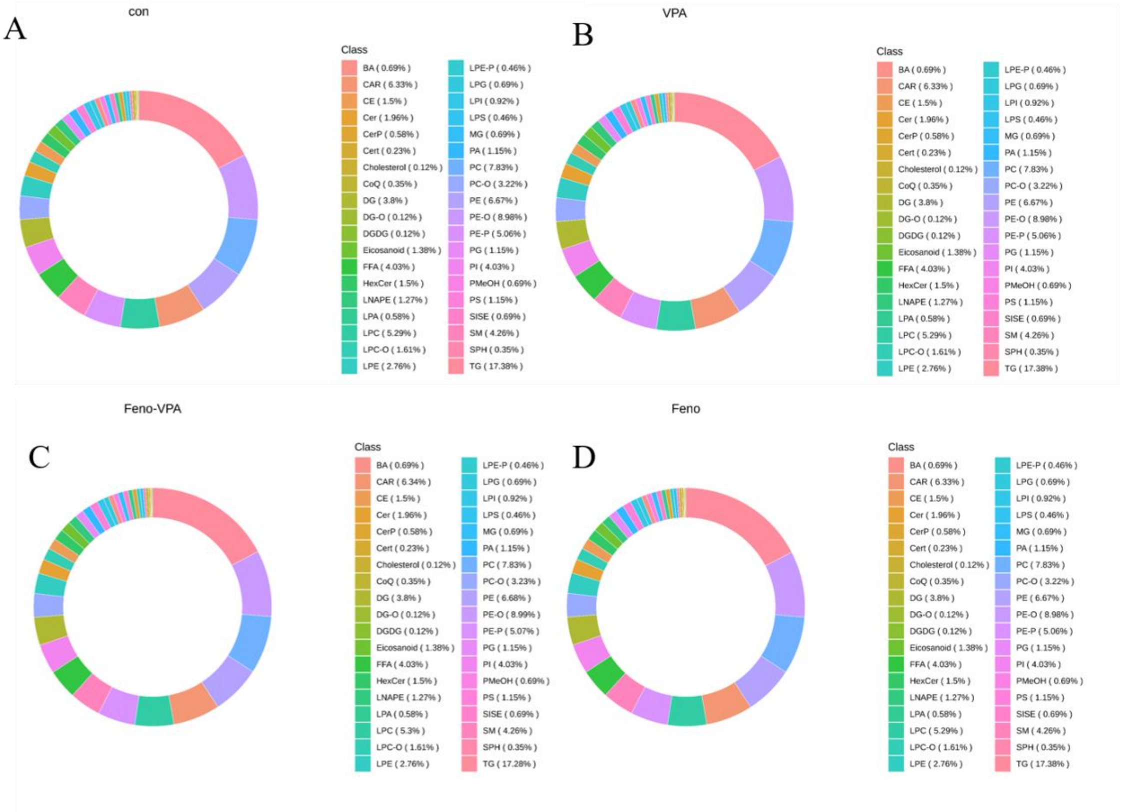

Figure S1 Ring diagram of lipid subclass composition for each group of samples.

Each color represents a lipid subclass, and the area of the color block indicates the proportion of that subclass. con: control group; VPA: VPA 500 mg/kg group; Feno: Feno50 mg/ kg group; VPA+Feno: VPA 500 mg/kg+ Feno50 mg/ kg group.

Table S1 Characteristics of control, NLF and ALF children

Parameters	Control	NLF	ALF
n	50	30	25
Age (years)	5.27±3.41	5.08±3.74	5.76±4.08
BMI（kg/cm^2）	18.96±4.29	18.92±3.88	19.42±4.35
Daily dose (mg/d)	-	409.23±264.08	414.36±213.86
Duration of treatment（month）	-	16.87±5.05	11.38±3.76
VPA concentration	-	57.74±28.25	59.91±28.10
TP	70.98±5.75	68.10±5.18	69.12±7.77
ALB	44.50±4.57	43.68±2.57	44.00±3.62
ALT	16.20±5.11	10.92±3.49	81.28±57.85*
AST	30.33±9.55	23.48±6.69	72.04±31.28*
ALP	217.00±44.40	216.48±51.56	363.13±220.51*
GGT	13.45±2.34	13.59±4.79	43.30±34.62*
TBIL	6.82±3.55	6.36±1.60	7.42±2.37

Data are means± SD; n, number of children. NLF: normal liver function; ALF: abnormal liver function; BMI: body mass index; VPA: VPA ; TP: total protein; ALB: albumin; ALT: alanine transaminase; AST: aspartate transaminase; ALP, alkaline phosphatasetotal bilirubin; GGT: glutamyltransferas; TBIL: total bilirubin *P<0.05

compared with control.

Table S2 List of real-time PCR Primers (Part 1)

Name	Forward primer (5'to 3')	Reverse primer (5'to 3')
PPARα(h)	CTTCGCAAACTTGGACCTGAA	GCTACCAGCATCCCGTCTTT
CPT1a(h)	AACCCAGAGTACGTGTCCAG	TTTCAGGTGCCTTCCAAAGC
VLCAD(h)	TCAGAGCATCGGTTTCAAAGG	AGGGCTCGGTTAGACAGAAAG
OCTN2(h)	AGGGACGATTTGAAGAGGCA	GTGGGACTGTTGCTTCTTGG
CACT(h)	CCTATCATCGGGGTCACTCC	AGCACTTGATCCGTTCTCCA
CPT2(h)	AGCCTGAGGAAAGTGGACTC	ACCCCAAGAGTGCTCAAAGT
β-actin(h)	CCTGGCACCCAGCACAAT	GGGCCGGACTCGTCATAC
GSTA2(h)	TTATGTCCCCCAGACCAAAG	CCTGTTGCCCACAAGGTAGT
GSTA4(h)	AGACCACGGAGAGGCT	CCTGACCACCTCAACATAGGG
GSTM3(h)	CCCCAACTTTGACCGAAGC	GGTGTCCATAACTTGGTTCTCCA
GPX1(h)	TGGACTGGTGGTGCTCG	CGTCACTGGGTGTTGGC
GPX2(h)	GGGCTGTGCTGATTGAGA	CGGACATACTTGAGGCTGTT
GPX3(h)	GGCTTCCCTTCCAACC	AATTTCTGCTCTTTCTCCC
GPX4(h)	ACGATGCCCACCCACT	CCACGCAGCCGTTCTT
CACT(h)	CCTATCATCGGGGTCACTCC	AGCACTTGATCCGTTCTCCA
OCTN2(h)	AGGGACGATTTGAAGAGGCA	GTGGGACTGTTGCTTCTTGG

Table S2 List of real-time PCR Primers (Part 2)

Name	Forward primer (5'to 3')	Reverse primer (5'to 3')
Pparα(m)	AGAGCCCCATCTGTCCTCTC	ACTGGTAGTCTGCAAAACCAAA
Octn2 (m)	CATCGCCAACTTCTCTGAGC	TTACACACCAGGTCCCACTC
Cact(m)	CCACAGGAATCATGACCCCT	CTTTGTAGAAGCCGCGGATC
Cpt2(m)	CGCCCAGCTTCCATCTTTAC	AACAAGTGTCGGTCAAAGCC
Cpt1a(m)	GGGCTACTCAGAGGATGGAC	AACTGGCACTGCTTAGGGAT
β-actin(m)	CACCATGTACCCAGGCATTG	CCTGCTTGCTGATCCACATC
Mcad (m)	CAACCTTCATCGCCATTTCT	GCCCAGAGAGCTCTAGACGA
Scad (m)	TTACCTGGCCTACTCCATCG	TGATCCACTGTTGCTTCTGC
Lcad (m)	GTCCGATTGCCAGCTAATGC	CACAGGCAGAAATCGCCAAC
Vlcad (m)	ATGGGAGAAGCAGGCAAACA	CACTGCCAGTTCACCACTGC
Cpt2 (m)	CGCCCAGCTTCCATCTTTAC	AACAAGTGTCGGTCAAAGCC
Cact (m)	CCACAGGAATCATGACCCCT	CTTTGTAGAAGCCGCGGATC
